# Dysarthria Speech Detection Using Convolutional Neural Networks with Gated Recurrent Unit

**DOI:** 10.3390/healthcare10101956

**Published:** 2022-10-07

**Authors:** Dong-Her Shih, Ching-Hsien Liao, Ting-Wei Wu, Xiao-Yin Xu, Ming-Hung Shih

**Affiliations:** 1 Department of Information Management, National Yunlin University of Science and Technology, Douliu 64002, Taiwan; 2 Department of Electrical and Computer Engineering, Iowa State University, 2520 Osborn Drive, Ames, IA 50011, USA

**Keywords:** dysarthria, deep learning, convolutional neural network, gated recurrent units

## Abstract

In recent years, due to the rise in the population and aging, the prevalence of neurological diseases is also increasing year by year. Among these patients with Parkinson’s disease, stroke, cerebral palsy, and other neurological symptoms, dysarthria often appears. If these dysarthria patients are not quickly detected and treated, it is easy to cause difficulties in disease course management. When the symptoms worsen, they can also affect the patient’s psychology and physiology. Most of the past studies on dysarthria detection used machine learning or deep learning models as classification models. This study proposes an integrated CNN-GRU model with convolutional neural networks and gated recurrent units to detect dysarthria. The experimental results show that the CNN-GRU model proposed in this study has the highest accuracy of 98.38%, which is superior to other research models.

## 1. Introduction

Speech is an essential medium of communication between people. Once the medium of communication is abnormal, it increases the difficulty of communication. Furthermore, many people with neurological diseases often have this condition, which is called dysarthria. Dysarthria is mainly a symptom caused by neuromuscular control disorders that affect breathing, vocalization, resonance, articulation, and prosody [1]. Sounds can be too loud or too low due to damage to the central and peripheral nervous system, such as stroke, Parkinson’s disease, brain trauma, brain tumors, cerebral palsy, amyotrophic lateral sclerosis, multiple sclerosis, muscular dystrophy, and other neurological diseases. The voice will also appear hoarse and lack tone changes. Hence, dysarthria patients are more likely to have abnormal speech characteristics [2].

Dysarthria may lead to social difficulties, a sense of isolation, and even world-weariness, depression, and other psychological problems [3]. Therefore, if there is no timely intervention and early rehabilitation training, it is easy to cause difficulties in disease course management, and the disease will continue to worsen. Doctors can subjectively diagnose dysarthria, but it is generally considered an expensive, laborious, and time-consuming test [4]. Therefore, having an objective and immediate automatic backing test is extremely important.

Deep learning has recently been popular and widely used in medical treatment. In order to objectively and accurately diagnose patients with dysarthria, more and more researchers are using deep learning to develop automatic detection of dysarthria. Many researchers use words for speech detection and different feature extraction methods to extract features from speech signals. For example, Vashkevich et al. [5] use pitch period entropy (PPE) based on acoustic features. Muhammad et al. [6] use glottal to noise excitation (GNE) and formant frequency or use spectrum and cepstrum for feature extraction. Other examples are mel-frequency cepstral coefficients (MFCC) [7], perception linear predictive coefficients (PLP), etc. [8]. After that, deep learning methods are used to detect dysarthria, such as convolutional neural network (CNN), CNN-LSTM (long short-term memory), and other models [9,10].

In the previous studies on the detection of dysarthria using the UA-Speech database, Narendra [10] selected the CNN-LSTM hybrid model as the classification model, but the accuracy of this model was only 77.57%. In order to improve the accuracy of the dysarthria detection model, this study used speech signals recorded by dysarthria patients and healthy people to undergo a short-time Fourier transform (STFT) and then convert the signals into spectrograms. After that, the signals were transformed into a spectral map, and mel-frequency cepstral coefficients (MFCC) were used to select the features. Finally, the accuracy in detecting dysarthria of the proposed CNN-GRU (gated recurrent unit) deep learning model was compared with three other models (CNN, LSTM, and CNN-LSTM).

## 2. Materials and Methods

### 2.1. Data Collection

Schlauch et al. [11] pointed out in their study that patients with dysarthria use words to make judgments with high recognition and low error rates. Therefore, this study chose words as the input audio samples for the subsequent studies. Our dataset was collected from the UA research database [12] (http://www.isle.illinois.edu/sst/data/UASpeech/, accessed on 18 February 2022). This database mainly contains the voice recordings of 15 dysarthria patients (4 women and 11 men) and 13 healthy subjects (4 women and 9 men), all of which were recorded by microphone and processed by noise removal. The subjects ranged in age from 18 to 58. A total of 455 words were recorded for each subject in the database, consisting of the numbers 1 to 10, the 26 letters, 19 computer command words, the 100 most common words from the Brown Corpus, and 300 words selected from the Project Gutenberg novel.

### 2.2. Method

**Figure 1:**
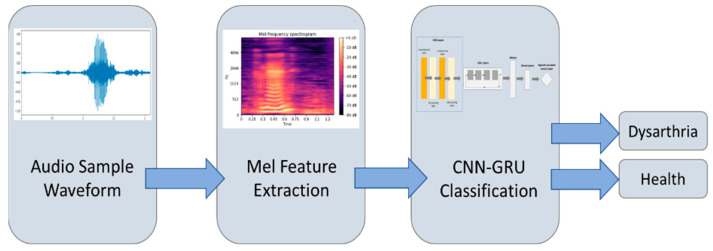
Flowchart of dysarthria detection.

The method proposed in this study consists of three stages, as shown in Figure 1. In the first stage, the original speech signal is transformed from the time domain to the frequency domain by a short-time Fourier transform. Second, the frequency domain data are extracted by mel-frequency cepstral coefficients. In the third stage, the features extracted from the mel spectrogram are used to detect and classify dysarthria patients and healthy people using the CNN-GRU model used in this study. In order to verify the excellence of the CNN-GRU deep learning model, this study also used the CNN model, LSTM model, and CNN-LSTM model to detect dysarthria and compare their results.

### 2.3. Data Preprocessing

**Figure 2:**
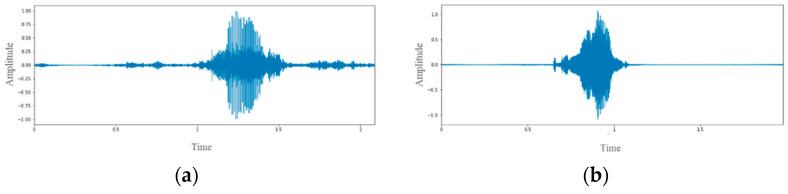
Amplitude waveforms of (**a**) dysarthria01 and (**b**) healthy01 subjects.

**Figure 3:**
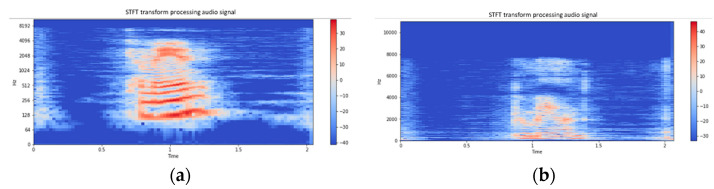
Short-time Fourier spectra of (**a**) dysarthria01 and (**b**) healthy01 subjects.

The audio could identify the amplitude waveform differences from the audio images of patients with dysarthria and healthy people through waveform images because people with dysarthria pronounce words more slowly and with a less steady pitch than healthy people. In general, the waveforms of the dysarthria patient (ID: dysarthria01) in Figure 2a are more irregular than the healthy subject (ID: healthy01) in Figure 2b. Audio waveforms can only show the relationship between amplitude and time. This study used Python Librosa to perform a short-time Fourier transform (STFT) of the audio. The short-time Fourier spectrograms of a dysarthria patient and a healthy subject are shown in Figure 3. From the spectrograms in Figure 3, it can be observed that the spectrum of subject dysarthria01 (Figure 3a) has more irregular frequencies and sudden higher decibels than the spectrum of subject healthy01 (Figure 3b).

Short-time Fourier transform (STFT) was used to transform speech signals from the time domain to the frequency domain. The frame length of the speech in this study was between 10 and 30 ms, the sampling frequency was set to 8 KHz, and the window length was set to 128 to improve the resolution. The transformation of the STFT voice signal x(T) to the frequency domain is shown in Equation (1).

(1) $$X\left(t,f\right)=\int_{-\infty }^{\infty }\omega \left(t-T\right)x\left(T\right)e^{-j2\pi fT}\;\mathrm{dT}\;$$

### 2.4. Feature Selection

Mel-frequency cepstral coefficients (MFCC) are widely used in speech recognition. Mel is the scale of tone frequencies picked up by the human ear. The relationship between the mel spectrum (M) and frequency (Hz) is shown in Equations (2) and (3).

(2) $$M\left(f\right)=2595\times \log_{10}\left(1+f/700\right)$$

(3) $$f=700\left(10^{\frac{m}{2595}}-1\right)$$

The power of spectrum, P(k), can be obtained from Equation (4).

(4) $$P\left(k\right)=\frac{1}{N}\;\;{\left|X\left(k\right)\right|}^{2}$$

The power of the spectrum, P(k), is passed through a series of mel-scale triangular filter windows to obtain the mel spectrum. The frequency, $H_{m}\left(k\right)$, of the triangular filter is calculated as shown in Equation (5).

(5) $$H_{m}\left(k\right)=\left\{\begin{array}{c} 0,\\ \frac{k-f\left(m-1\right)}{f\left(m\right)-f\left(m-1\right)}\\ \frac{f\left(m+1\right)-k}{f\left(m+1\right)-f\left(m\right)},\\ 0, \end{array}\right.\;,$$

*f(m)* is the central frequency of the mel triangle filter. The logarithmic energy spectrum of each frame is S(m), which is obtained using a logarithmic process, as shown in Equation (6).

(6) $$S\left(m\right)=\ln[\sum \begin{array}{c} N-1\\ K=0 \end{array}P\left(k\right)H_{m}\left(k\right)],0\leq m\leq M$$

$P\left(k\right)$ is the power spectrum, $H_{m}\left(k\right)$ is the filter window, and *M* is the number of filter windows.

**Figure 4:**
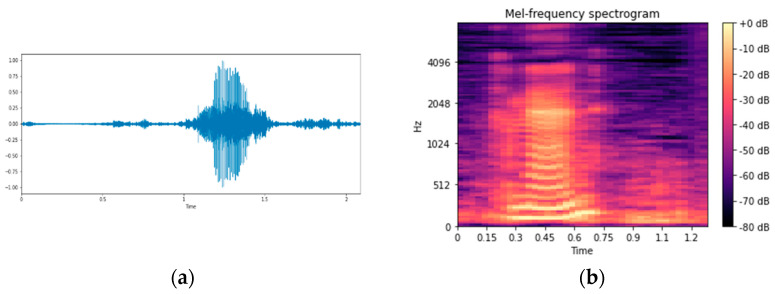
Feature extraction: (**a**) voice signal and (**b**) mel spectrum.

This study used Librosa in Python software to extract the feature of the inverse coefficient of the mel frequency. Figure 4a is the voice signal. After extracting the speech signal samples and features, the mel spectrum is shown in Figure 4b.

### 2.5. Deep Learning Algorithms

#### 2.5.1. CNN Model

**Figure 5:**
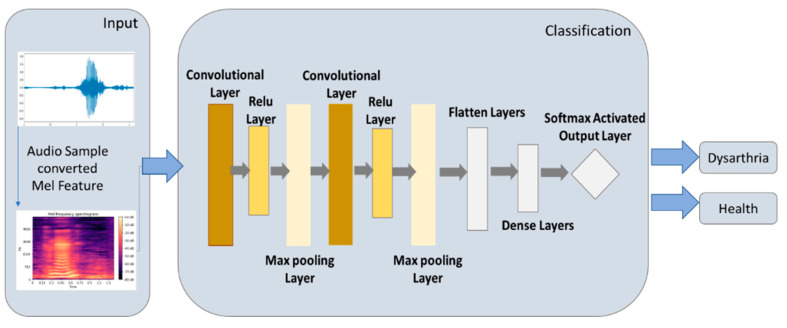
Architecture of CNN model.

The CNN model can be used to detect the critical features of the audio in the audio message [13]. The CNN model’s output and input architecture is shown in Figure 5, and the core CNN model is explained as follows.

The CNN model uses the convolution layer to retain the original feature arrangement of the image and obtain some essential features from the image. Then, the max pooling layer is used to select the more intense feature values from the essential features and shave the weak ones. This study adopted a rectified linear unit (Relu) to shave off the eigenvalues less than 0 at the site to speed up model training between the convolution layer and the max pooling layer. Then, the feature values are converted into one-dimensional data through the flatten layer to facilitate the subsequent use of the fully connected layer. Finally, the activation function of softmax is connected to the classification output. Table 1 shows the parameter settings of the CNN model in this study.

#### 2.5.2. LSTM Model

**Figure 6:**
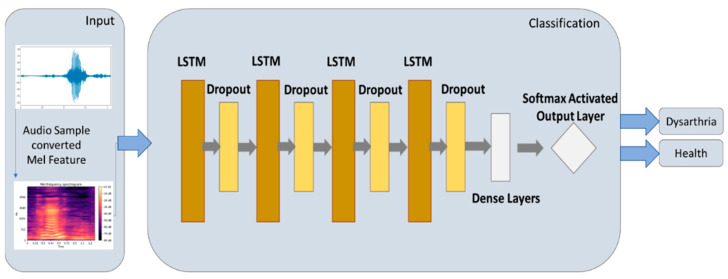
Architecture of LSTM model.

Speech is a typical temporal signal because the LSTM (long short-term memory) model has a solid temporal ability [14]. The output and input architecture of the LSTM model is shown in Figure 6. In this study, a four-layer LSTM was used as the input layer, and a four-layer dropout was added to prevent the over-fitting problem of the model in the training process. A dense layer was used for dimensional transformation, and softmax was used for the classification output. Table 2 shows the parameter settings of the LSTM model in this study.

#### 2.5.3. CNN-LSTM Model

**Figure 7:**
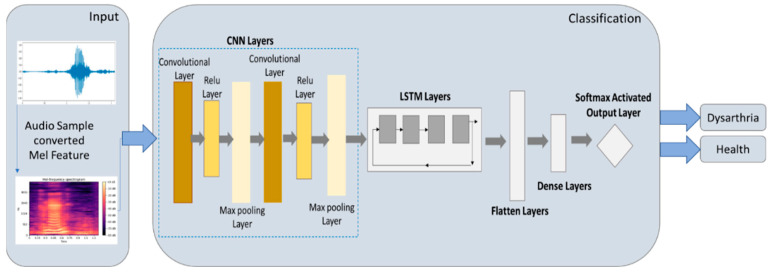
Architecture of CNN-LSTM model.

CNN combined with LSTM for speech detection is an efficient and accurate hybrid model [15]. The CNN-LSTM model uses a CNN convolution layer to retain the original feature arrangement of an image and obtain some essential features from the image. Then, the max pooling layer is used to select the more intense feature values from the essential features and shave the weak ones. Between the convolution layer and the max pooling layer, the rectified linear unit (Relu) is provided to shave off feature values less than 0 to speed up model training. Then, the LSTM is connected to capture the temporal dynamics of the sequence, and the flatten layer is connected to convert the feature values into one-dimensional data. Finally, the activation function of softmax is connected for classification output. The output and input architecture of the CNN-LSTM model is shown in Figure 7. Table 3 shows the parameter settings of the CNN-LSTM model in this study.

#### 2.5.4. CNN-GRU Model

**Figure 8:**
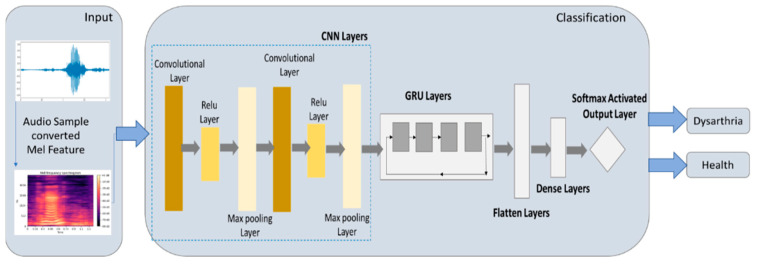
Architecture of CNN-GRU model.

CNN combined with GRU was used as a classifier in the study of speech enhancement [16] and android botnet detection [17]. In GRU architecture, fewer parameters need to be set, and it is simpler than LSTM architecture [18]. Therefore, it becomes natural to use GRU to optimize the CNN model. However, the combination of CNN and GRU is not always the same. This study combines the studies by Hasannezhad et al. [16] and Yerima et al. [17] into a different CNN-GRU model and writes programs through Python’s Keras package for experiments. The output and input architecture of the CNN-GRU model proposed in this study is shown in Figure 8.

The CNN-GRU model proposed in this study uses a CNN convolutional layer to retain the original feature arrangement of an image and obtains some essential features from the image. In addition, the max pooling layer is used to select more intense feature values from important features and shave the weak feature values, which can prevent the problem of over-fitting the model. Between the convolutional layer and the max pooling layer, this study also used a rectified linear unit to shave off the eigenvalues less than 0 to accelerate model training. Then, the eigenvalues are passed through the update gate and reset gate of the gated recurrent unit (GRU) to increase the calculation speed of the model so that the model can be more accurate. Then, the flattened layer is connected to convert the feature value into one-dimensional data, which is convenient for the subsequent use of the fully connected layer. Finally, softmax’s activation function is connected as the output to determine whether the speech audio is dysarthria. Table 4 shows the parameter settings of the CNN-GRU model in this study.

### 2.6. Experimental Design

In this study, the word audio of dysarthria patients and healthy subjects was converted into the frequency domain by short-time Fourier transformation, and the mel spectrum image was extracted by the mel-frequency cepstral coefficient as the input of the four models, including CNN, LSTM, CNN-LSTM, and CNN-GRU, proposed in this study. The pros and cons of each model of dysarthria detection were compared by the training and validation sets and the test results. The dataset was divided into the training set, validation set, and test set, which were used for the training and testing of the four deep learning models. The distribution of data was based on the ratio of 0.7:0.15:0.15. In this study, after comprehensive area testing, these parameters had different batch sizes and learning rates to obtain the ideal solution. In the case of batch sizes of 32, 64, and 128, learning rates of 0.01, 0.001 and 0.0001, and Epoch = 10, the experimental results are described in detail in Section 3.

### 2.7. Model Evaluation

In this study, the effectiveness of the deep learning models was evaluated by the following evaluation indicators, which are generally divided into four types: (1) true positive (TP); (2) true negative (TN); (3) false positive (FP); and false negative (FN). The following evaluation metrics of the models can be calculated and learned based on those four results: accuracy, precision, recall, f1-score, and ROC curve [19,20].

## 3. Experimental Results

### 3.1. Experimental Results of CNN Model

**Figure 9:**
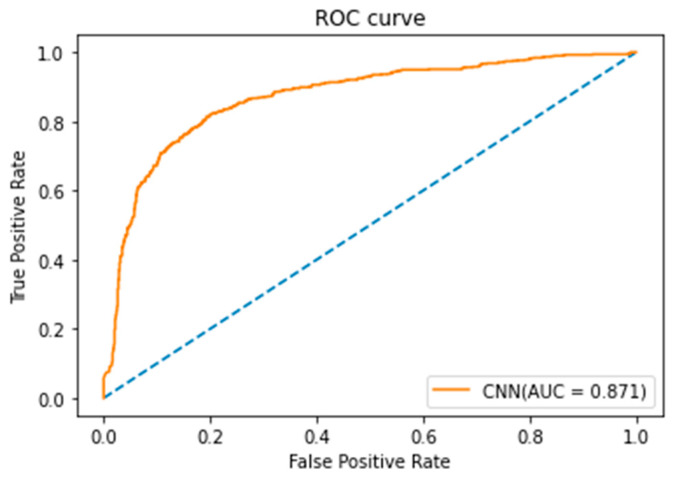
ROC curve of CNN model.

The CNN model in this study adopted Keras in Python software for model training [21], and the classification results of the CNN model are shown in Table 5. If the CNN model parameter value of the batch size was set as 128 and the learning rate was set as 0.01, the highest accuracy of 94.36% of the CNN model could be obtained. In this study, Scikit-Learn [20] in Python software was used to draw the ROC curve of the CNN model in Figure 9, from which it can be seen that the AUC of the CNN model was 0.871 and the classification result of the model was good.

In this study, the epoch parameter value of the CNN model was set as 10, and the execution time, loss function, and accuracy of the CNN model can be observed from the training process in Table 6. The accuracy of the test set was 94.36%. It only took about 3 ms/epoch to train the CNN model. The accuracy of the final training set was 97.88%, and the loss function was 0.0638.

### 3.2. Experimental Results of LSTM Model

**Figure 10:**
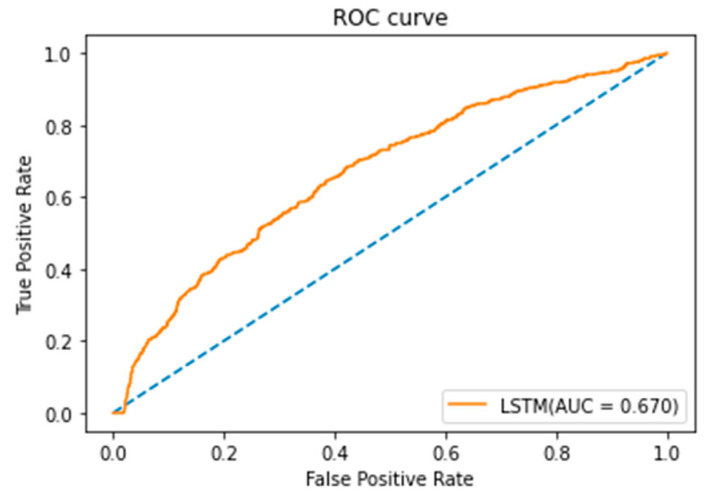
ROC curve of LSTM model.

The LSTM model in this study adopted Keras in Python software for model training [21]. The classification results of the LSTM model are shown in Table 7. In the LSTM model, if the parameter value of the batch size was set as 64 and the learning rate was set as 0.001, the LSTM model could achieve the highest accuracy of 56.61%. In this study, Scikit-Learn [22] in Python software was used to draw the ROC curve of the LSTM model in Figure 10, from which it can be seen that the AUC of the LSTM model was 0.670 and the classification result of the model was, in general, poor. According to the area under an ROC curve (https://darwin.unmc.edu/dxtests/roc3.htm, accessed on 27 September 2022), AUC is divided into five grades: 0.9–1 = excellent (A), 0.80–0.90 = good (B), 0.70–0.80 = fair (C), 0.60–0.70 = poor (D), and 0.50–0.60 = fail (F). Since the AUC result of LSTM was 0.67, the effect of LSTM was considered poor in this study.

In this study, the epoch parameter values of the LSTM model were set as 10, and the execution time, loss function, and accuracy of the LSTM model can be observed from the training process data in Table 8. The training time of the LSTM model was only about 2 ms/epoch. The accuracy of the final training set was 56.60%, and the loss function was 0.7562. The accuracy of the test set was 56.61%.

### 3.3. Experimental Results of CNN-LSTM

**Figure 11:**
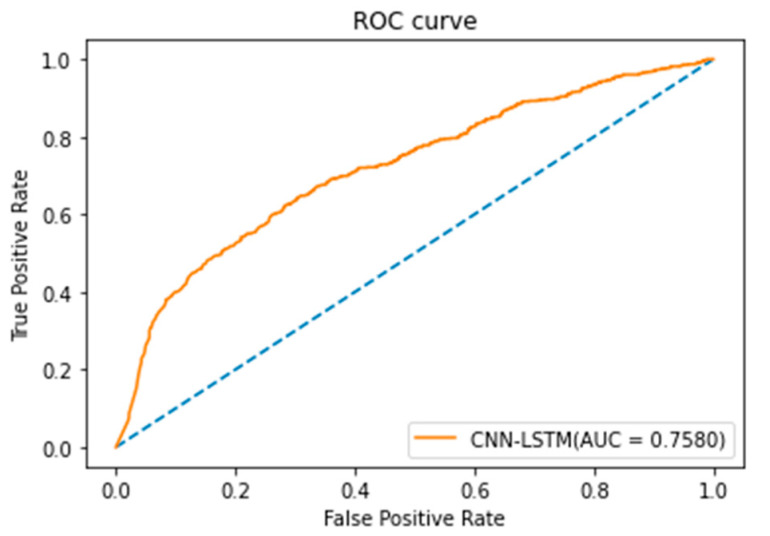
ROC curve of CNN-LSTM model.

The CNN-LSTM model in this study adopted Keras in Python software for model training [21]. The classification results of the CNN-LSTM model are shown in Table 9. In the CNN-LSTM model, if the parameter value of the batch size was set as 128 and the learning rate was set as 0.01, the CNN-LSTM model could obtain the highest accuracy of 78.57%. In this study, Scikit-Learn [22] in Python software was used to draw the ROC curve of the CNN-LSTM model in Figure 11. It can be seen from Figure 11 that the AUC of the CNN-LSTM model was 0.758 and the classification result of the model was above medium.

In this study, the epoch parameter value of the CNN-LSTM model was set as 10, and the execution time, loss function, and accuracy of the CNN-LSTM model can be observed from the training process data in Table 10. The training time of the CNN-LSTM model was only about 4 to 8 ms/epoch, and the accuracy of the final training set was 84.21%. The loss function was 0.2745, and the test set accuracy was 78.57%.

### 3.4. Experimental Results of CNN-GRU Model

**Figure 12:**
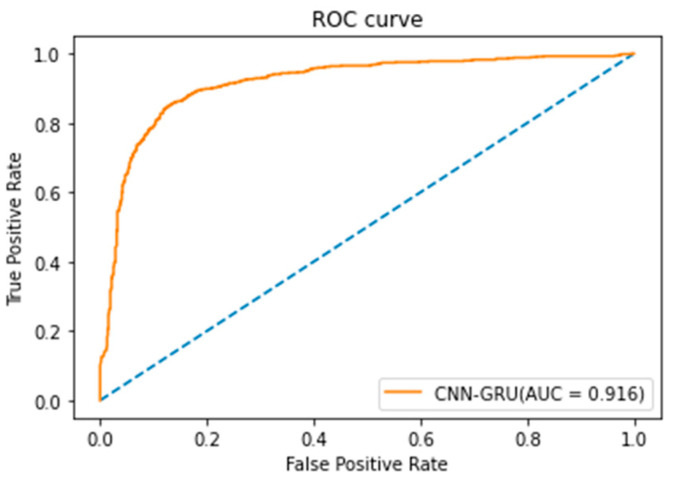
ROC curve of CNN-GRU model.

The CNN-GRU model in this study adopted Keras in Python software for model training [21]. The classification results of the CNN-GRU model are shown in Table 11. In the CNN-GRU model, if the parameter value of the batch size was set as 128 and the learning rate was set as 0.001, the highest accuracy of 98.88% of the CNN-GRU model could be obtained. In this study, Scikit-Learn [22] in Python software was used to draw the research results of the ROC curve of the CNN-GRU model in Figure 12. It can be seen that the AUC of the CNN-GRU model was 0.916 and the model classification results were excellent.

In this study, the epoch parameter value of the CNN-GRU model was set as 10, and the execution time, loss function, and accuracy of the CNN-GRU model can be observed from the training process data in Table 12. It only took about 2 ms/epoch to train the CNN-GRU model, and the accuracy of the final training set was 98.14%. The loss function was 0.1621, and its test set accuracy was 98.38%.

## 4. Discussion of Results

According to the experimental results in Section 3, the accuracy values of the CNN model training set and test set were 97.88% and 94.36%, respectively (Table 6). The accuracy of the LSTM model training set and test set was 56.61% (Table 8). The accuracy of the CNN-LSTM model training set was 84.21%, and the accuracy of the test set was 78.57% (Table 10). Finally, the accuracy values of the proposed CNN-GRU model training set and test set were 98.14% and 98.38%, respectively (Table 12). Regardless of the perspective of the training set and test set, the CNN-GRU model had the highest accuracy. In the judgment of the AUC value, the AUC = 0.916 of the CNN-GRU model was also the highest, which was better than the other three models. Various evaluation metrics show that the proposed CNN-GRU model can obtain more accurate judgment results in dysarthria detection.

The results of this study are compared with other methods used in previous studies and summarized in Table 13. Hernandez et al. [23] used a method based on fricative sounds in audio messages and machine learning to detect dysarthria. The average spectral peak in the spectral moment was used to extract the fricatives in the audio as the input features of the SVM model, and the final SVM accuracy was 72%. Narendra et al. [24] trained an SVM with acoustic and glottic features extracted from coded speech utterances and their corresponding dysarthria/health labels and finally achieved an accuracy of 96.38% from the SVM. Narendra et al. [25] developed an end-to-end system that mainly used raw speech signals and raw glottal flow waveforms to detect dysarthria in two deep learning architectures: CNN-MLP and CNN-LSTM. The results showed that the original glottal flow waveform is more suitable for model training than the original speech signal, and the accuracy of CNN-MLP and CNN-LSTM were 87.93% and 77.57%, respectively. Rajeswari et al. [26] enhanced the speech by variational mode decomposition and fed the reconstructed signal to CNN for model training, and the final result achieved 95.95% accuracy. The accuracy of the CNN-GRU model proposed in this study was 98.38%, which is the highest in all studies. However, our approach may take a longer time to execute. After a survey, previous studies have not reported the execution times in their articles. Therefore, an execution time of 2 ms for our approach is appended in Table 13 for further investigation or comparison in the future.

## 5. Conclusions

Although dysarthria testing can be based on the subjective judgment of doctors, it is also regarded as a costly and time-consuming test, which can easily cause a medical burden. Therefore, if dysarthria testing can be conducted objectively, it can assist doctors in making an immediate judgment. This study used a CNN-GRU classification model for dysarthria detection. The results showed that the proposed CNN-GRU model can achieve the highest accuracy of 98.38%, which is better than the CNN, LSTM, CNN-LSTM models and those of other scholars.

The results can be used as an auxiliary diagnostic procedure for detecting dysarthria in the future. In future studies, it may be possible to take more eigenvalues from audio to analyze the severity level of dysarthria symptoms so that dysarthria detection can be further studied. In addition, others can also use the CNN-GRU model to detect other speech pathologies, such as Parkinson’s disease, amyotrophic lateral sclerosis (ALS), and other symptoms of speech detection. The proposed architecture can also be used for image identification, just as Priyanka and Ganesan [26] used different data preprocessing methods combined with machine learning to classify the severity of dementia. Better prediction results may be achieved if the research is conducted through deep learning architecture.

In addition, most of the existing freely available dysarthric speech databases, including [12], contain speech data recorded from a small number of patients [24]. The volume of speech samples recorded in the dataset used in this study is quite large. However, the number of samples included is not immense, and there has been no continuous addition of samples, which makes it challenging to ensure that the results of this study can be adequately transferred to other clinical trials of dysarthria.

## Figures and Tables

**Table 1:** Parameters of CNN model.

Network	Layer	No. of Activations	No. of Parameters
CNN	Cov1	(27,27,32)	160
	Maxpooling1	(13,13,32)	0
	Cov2	(12,12,64)	8256
	Maxpooling2	(6,6,64)	0
	Flatten	(2304)	0
	Dense	(3)	387

**Table 2:** Parameters of LSTM model.

Network	Layer	No. of Activations	No. of Parameters
LSTM	LSTM	(26,10)	480
	Dropout	(26,10)	0
	LSTM	(26,10)	840
	Dropout	(26,10)	0
	LSTM	(26,10)	840
	Dropout	(26,10)	0
	LSTM	(26,10)	840
	Dropout	(26,10)	0
	Dense	(26,2)	22

**Table 3:** Parameters of CNN-LSTM model.

Network	Layer	No. of Activations	No. of Parameters
CNN-LSTM	Cov1	(23,32)	320
	Maxpooling1	(11,32)	0
	Cov2	(11,64)	14,400
	Maxpooling2	(5,64)	0
	LSTM	(2,128)	20,608
	Flatten	(64)	0
	Dense	(44)	2860

**Table 4:** Parameters of CNN-GRU model.

Network	Layer	No. of Activations	No. of Parameters
CNN-GRU	Cov1	(23,32)	320
	Maxpooling1	(11,32)	0
	Cov2	(11,64)	14,400
	Maxpooling2	(5,64)	0
	GRU	(2,32)	15,552
	Flatten	(64)	0
	Dense	(44)	2860

**Table 5:** Classification results of CNN model.

Model	Batch Size	Learning Rate	Accuracy%	Precision%	Recall	F1-Score
CNN	32	0.1	70.00	70.01	0.7002	0.7010
		0.01	88.89	69.44	0.8334	0.7575
		0.001	75.00	75.50	0.7500	0.7506
	64	0.1	88.88	86.66	0.8333	0.8148
		0.01	89.89	87.50	0.8333	0.8285
		0.001	86.67	79.99	0.666	0.6249
	128	0.1	93.33	87.50	0.8333	0.8285
		0.01	94.36	90.39	0.8913	0.8896
		0.001	94.35	86.66	0.8333	0.8148

**Table 6:** Execution time, loss function, and accuracy of CNN model.

Epoch	Execution Time (ms)	Accuracy (Training) (%)	Loss Function	Accuracy (Validation) (%)	Accuracy (Testing) (%)
1	3	82.23	0.4972	45.40	79.20
2	3	91.26	0.2129	83.41	90.27
3	3	93.42	0.1674	90.35	91.27
4	3	94.18	0.1444	92.64	92.30
5	3	94.86	0.1314	93.90	93.24
6	3	95.77	0.1134	94.87	93.50
7	3	96.60	0.0931	95.48	93.52
8	3	96.52	0.0894	96.37	94.27
9	3	97.21	0.0757	97.13	94.20
10	3	**97.88**	**0.0638**	**97.53**	**94.36**

**Table 7:** Classification results of LSTM model.

Model	Batch Size	Learning Rate	Accuracy%	Precision%	Recall	F1-Score
LSTM	32	0.1	50.32	50.10	0.5001	0.5002
		0.01	54.29	53.21	0.5321	0.5321
		0.001	54.67	54.60	0.5460	0.5430
	64	0.1	55.60	44.21	0.4421	0.4421
		0.01	54.30	54.12	0.5420	0.5411
		0.001	56.61	53.43	0.5435	0.5324
	128	0.1	55.21	44.25	0.6550	0.5220
		0.01	56.60	43.21	0.5521	0.4201
		0.001	55.37	50.20	0.5020	0.5020

**Table 8:** Execution time, loss function, and accuracy of LSTM model.

Epoch	Execution Time (ms)	Accuracy (Training) (%)	Loss Function	Accuracy (Validation) (%)	Accuracy (Testing) (%)
1	2	53.32	0.7346	54.89	53.20
2	2	53.63	0.7360	55.32	53.39
3	2	54.29	0.7375	55.36	53.65
4	2	54.42	0.7394	54.22	54.30
5	2	54.68	0.7153	55.89	54.39
6	2	54.56	0.7163	55.90	54.37
7	2	54.04	0.7309	55.91	54.49
8	2	55.60	0.7316	56.01	55.60
9	2	56.01	0.7557	56.43	55.97
10	2	**56.60**	**0.7562**	**56.42**	**56.61**

**Table 9:** Classification results of CNN-LSTM model.

Model	Batch Size	Learning Rate	Accuracy%	Precision%	Recall	F1-Score
CNN-LSTM	32	0.1	62.49	65.99	0.6666	0.6549
		0.01	66.66	64.44	0.6656	0.6333
		0.001	73.20	68.54	0.6756	0.6723
	64	0.1	70.21	69.45	0.6230	0.7165
		0.01	69.20	69.44	0.7333	0.7175
		0.001	73.21	67.54	0.6740	0.6740
	128	0.1	75.30	70.15	0.6563	0.7490
		0.01	78.57	70.33	0.6660	0.7500
		0.001	77.33	69.44	0.7475	0.7375

**Table 10:** Execution time, loss function, and accuracy of CNN-LSTM model.

Epoch	Execution Time (ms)	Accuracy (Training) (%)	Loss Function	Accuracy (Validation) (%)	Accuracy (Testing) (%)
1	5	42.11	0.9493	50.00	43.50
2	5	57.89	0.8010	66.67	50.65
3	5	63.16	0.6720	66.67	51.27
4	8	73.68	0.5617	66.67	65.90
5	4	84.21	0.3367	83.33	66.37
6	8	84.21	0.3256	83.33	67.47
7	5	84.21	0.3102	83.33	70.30
8	6	84.21	0.3060	83.33	75.98
9	5	84.21	0.2665	83.33	76.35
10	5	**84.21**	**0.2745**	**83.33**	**78.57**

**Table 11:** Classification results of CNN-GRU model.

Model	Batch Size	Learning Rate	Accuracy%	Precision%	Recall	F1-Score
CNN-GRU	32	0.1	92.27	93.21	0.9121	0.9220
		0.01	94.52	94.23	0.9422	0.9420
		0.001	95.21	93.20	0.9220	0.9231
	64	0.1	96.41	95.51	0.9421	0.9412
		0.01	96.70	90.24	0.9026	0.9633
		0.001	96.38	96.31	0.9427	0.9532
	128	0.1	97.71	96.21	0.9621	0.9621
		0.01	98.02	90.47	0.9030	0.9021
		0.001	98.88	91.47	0.9147	0.9147

**Table 12:** Execution time, loss function, and accuracy of CNN-GRU model.

Epoch	Execution Time (ms)	Accuracy (Training) (%)	Loss Function	Accuracy (Validation) (%)	Accuracy (Testing) (%)
1	2	79.20	0.157	90.77	89.21
2	2	92.27	0.1267	91.08	90.20
3	2	94.52	0.3353	90.97	93.45
4	2	95.21	0.2937	91.16	94.60
5	2	96.41	0.1553	90.77	95.88
6	2	96.70	0.1274	91.36	97.56
7	2	96.83	0.1029	91.20	96.30
8	2	97.71	0.2396	91.40	97.13
9	2	98.02	0.2084	91.63	97.79
10	2	**98.14**	**0.1621**	**91.52**	**98.38**

**Table 13:** Performance comparison.

Author	Classification Method	Dataset	Accuracy (%)	Execution Time
Hernandez et al. (2019) [23]	SVM	UA-Speech	72%	-
Narendra et al. (2019) [24]	SVM	UA-Speech	96.38%	-
Narendra et al. (2020) [10]	CNN-MLP	UA-Speech	87.93%	-
	CNN-LSTM		77.57%	-
Rajeswari et al. (2022) [25]	CNN	UA-Speech	95.95%	-
Our Approach	CNN-GRU	UA-Speech	98.38%	2 ms

(-: indicates unknown or uncertainty).

## Data Availability

Not applicable.
